# The Role of Neuroinflammation in Post-traumatic Epilepsy

**DOI:** 10.3389/fneur.2021.646152

**Published:** 2021-05-28

**Authors:** Lei Sun, Wei Shan, Huajun Yang, Ru Liu, Jianping Wu, Qun Wang

**Affiliations:** ^1^Beijing Tiantan Hospital, Capital Medical University, Beijing, China; ^2^Advanced Innovation Center for Human Brain Protection, Capital Medical University, Beijing, China; ^3^National Center for Clinical Medicine of Neurological Diseases, Beijing, China; ^4^Beijing Friendship Hospital, Capital Medical University, Beijing, China; ^5^Beijing Institute for Brain Disorders, Beijing, China

**Keywords:** post-traumatic epilepsy, traumatic brain injury, neuroinflammation, epileptogenesis, immunotherapy

## Abstract

Post-traumatic epilepsy (PTE) is one of the consequences after traumatic brain injury (TBI), which increases the morbidity and mortality of survivors. About 20% of patients with TBI will develop PTE, and at least one-third of them are resistant to conventional antiepileptic drugs (AEDs). Therefore, it is of utmost importance to explore the mechanisms underlying PTE from a new perspective. More recently, neuroinflammation has been proposed to play a significant role in epileptogenesis. This review focuses particularly on glial cells activation, peripheral leukocytes infiltration, inflammatory cytokines release and chronic neuroinflammation occurrence post-TBI. Although the immune response to TBI appears to be primarily pro-epileptogenic, further research is needed to clarify the causal relationships. A better understanding of how neuroinflammation contributes to the development of PTE is of vital importance. Novel prevention and treatment strategies based on the neuroinflammatory mechanisms underlying epileptogenesis are evidently needed.

**Search Strategy:**

Search MeSH Terms in pubmed: “[“Epilepsy”(Mesh)] AND “Brain Injuries, Traumatic”[Mesh]”. Published in last 30 years. 160 results were founded. Full text available:145 results. Record screened manually related to Neuroinflammation and Post-traumatic epilepsy. Then finally 123 records were included.

## Introduction

Epilepsy is a chronic neurological disease that is characterized by recurrent, transient and episodic discharge of neurons in the brain. The recurrent and frequent seizures seriously affect patients' life quality and cause a substantial economic burden to society and family (1). Post-traumatic seizure (PTS) is one of the severe consequences of brain trauma, with an incidence ranging from 4 to 53% (2, 3). According to different latency from injury to seizure onset, PTS can be divided into immediate (<24 h), early (1–7 d), or late (>1 week) seizures, and only the recurrent late seizures can be called post-traumatic epilepsy (PTE) (4, 5). Therefore, PTE can be generally defined as unprovoked and recurrent seizures that occur more than 1 week after traumatic brain injury (TBI), accounting for as high as 20% of acquired epilepsy and 4% of all patients with epilepsy (6). Injury severity, age, and surgical methods after trauma are important risk factors for developing PTE. Moreover, we should also consider other factors such as hypoxia, hyperthermia, intracerebral bleeding, infection, or status epilepticus (SE) combining TBI that may increase PTE risk.

The pathogenesis of PTE is not yet clear. A growing body of evidence from clinical and experimental studies suggested the involvement of neuroinflammation in the process of epileptogenesis post TBI (7–9). Acute and early epileptic attacks may be a direct response to brain injury: Epidural and subdural hematoma, cerebral edema and brain contusion occur at the time of head impact can compress and stimulate the focal damaged tissue, which may cause blood-brain barrier (BBB) breakdown and reduce the threshold of seizure (10, 11). In contrast, the late onset is mediated by several factors including, but not limited to: generation of oxygen free radicals, abnormal release of excitotoxicity neurotransmitters, neuroimmune abnormalities caused by the inflammatory response, and neural network remodeling consisting of neurogenesis and neurodegeneration. The complexity of mechanisms and the severity of injury are the leading causes of the different outcomes and prognosis of PTE patients. Neuroinflammation is a crucial component of the epileptogenesis following TBI, and is also a promising target for treatment. Since neuroinflammatory mechanisms can be harmful or beneficial, it is necessary to have a good understanding of the timing and complexity of the immune response after TBI before developing immunomodulatory therapies to develop new preventative treatments of PTE.

## Neuroinflammation Secondary To TBI Driving PTE

**Figure 1:**
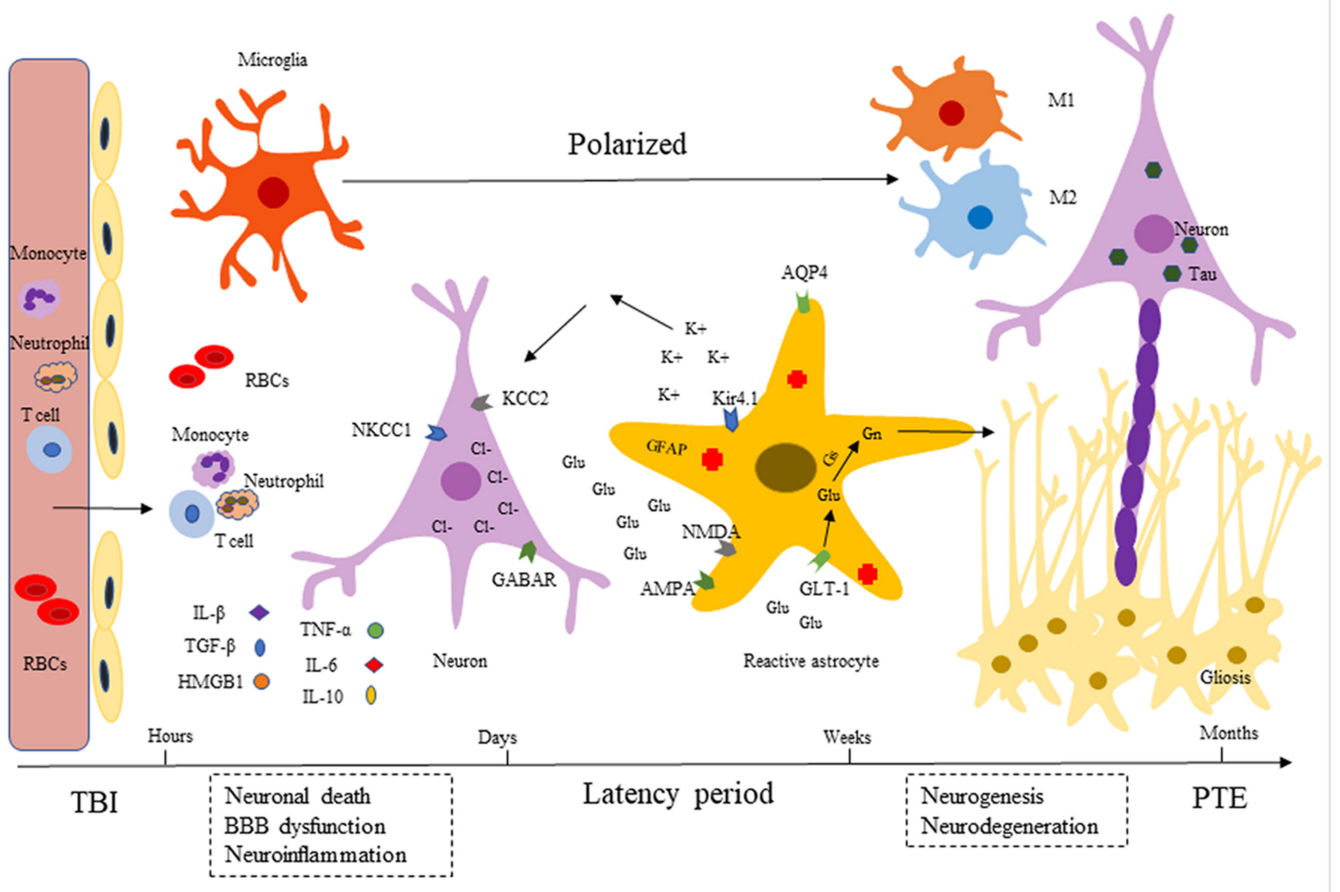
Development of post-traumatic epilepsy (PTE). After TBI, there are a series of immune inflammatory reactions including BBB dysfunction (endothelial cells destruction), microglia and astrocytes activation, neuroinflammatory factors release (IL-1β, HMGB1 TGF-β, TNF-α, etc.). Released inflammatory cytokines can recruit neutrophils and monocytes into injured tissues, expanding the inflammatory cascade. Over time (months to years), injury-induced neurogenesis and neuroplasticity help repair and regeneration, persistent chronic neuroinflammation promotes neurodegeneration (Tau accumulation). These pathological processes lead to excessive excitement of neurons, which eventually causes repeated seizures.

TBI is one of the common emergencies in neurosurgery with high rates of mortality and disability. There is increasing evidence that TBI can cause direct and immediately impacts and evolves over time, contributing to long-term sequelae, such as behavioral disturbances, epilepsy and neurodegenerative disorders (12). The pathological mechanisms are characterized by a robust immune response, including BBB damage, activation of glial cells, infiltration of peripheral leukocytes, and release of pro- and anti-inflammatory cytokines (IL-1β, HMGB1 TGF-β, TNF-α, etc.). Over time, from months to years, neurogenesis and neuroplasticity caused by injury help repair and regeneration, and an ongoing chronic neuroinflammation promotes neurodegeneration. These pathological processes lead to excessive excitation of neurons and ultimately drive PTE development (Figure 1).

The immune system is considered to react to injury with a two-phase response: innate immunity and adaptive immunity, the latter characterized by antigen-specificity and “remember” ability, which plays a vital role in our defense against pathogens. Innate immune response secondary to damaged or infected central nervous system (CNS) is mediated partly by damage- or pathogen-associated molecule patterns (DAMPs and PAMPs) (13). By interacting with “danger” sensors-pattern recognition receptors (PRRs), such as toll-like receptors (TLRs), nucleotide-binding oligomerization domain (NOD) like receptors (NLRs), and scavenger receptors, pathogens and danger signals can act to initiate the innate immune response. Innate immune cells (e.g., phagocytes, granulocytes and T lymphocytes et al) can then release cytokines and chemokines that amplify the inflammatory cascade. Adaptive (or acquired) immunity is a more targeted response, which develop immunological memory to a specific pathogen in an initial response. CD8 and CD4 T lymphocytes recognize major histocompatibility complex (MHC) and play cytotoxic, helper or regulatory roles. On the other hand, B lymphocytes can produce immunoglobulins and participate in T cell activation (14).

Inflammasome, a multi-protein complex assembled by cytosolic receptors, is an essential component of the innate immune system and upon activation can be involved in the production of pro-inflammatory cytokines. NLRP1, NLRP3, NLRC4, and AIM2 are the most concerned inflammasomes in brain. Inflammasomes, especially NLRP3, can recognize DAMPs or PAMPs, recruit and activate pro-inflammatory protease Caspase-1. Activated Caspase-1 cleaves the precursors of IL-1β and IL-18 into their mature forms, which plays a vital role in promoting sterile immune response following TBI (15, 16). In addition to inducing cytokine release, activation of inflammasomes can also mediate pyroptosis, a form of necrotic cell death (17). Adamczak et al. (18) found that inflammasome components caspase-1, ASC and NLRP-1 were significantly elevated in the cerebrospinal fluid (CSF) of patients with moderate or severe brain trauma, and the levels of these proteins were correlated with unfavorable outcomes. One study showed that administration of anti-ASC antibodies in a mouse of fluid-percussion injury (FPI) model reduced capase-1 activation and IL-1β generation, resulting in a decrease in brain lesion volume. These data indicate that inflammasome proteins might serve as potential biomarkers to assess inflammation and TBI severity.

### Microglial Activation

Microglia are sentinel cells of the CNS and are often the first responders to brain damage. Under physiological conditions, microglia are resting, capable of sensing inflammatory signals, promoting neuronal survival and synaptic remodeling, thus playing an “immune surveillance” role. After TBI, microglial cells can be activated rapidly and sustained for several years in the brain (19). Microglia response to brain trauma has several phases, including morphological transformation, proliferation, migration, phagocytosis and chemokines or cytokines release (19, 20). After acute classical activation, microglia can release various inflammatory mediators, including IL-1β, IL-6, TNF-α, nitric oxide (NO), metalloproteinases, and reactive oxygen species (ROS). The production of these inflammatory mediators can promote the immune response to TBI by increasing BBB permeability and facilitating peripheral immune cell recruitment. The researchers found that minocycline specifically inhibited the activation of microglia and monocytes, which could reduce the volume of trauma foci and improve the neurological outcome of the mice (21). However, it is worth noting that microglia are highly plastic cells, and how they promote sterile immune response also rely on their activation state, type of damage, and interaction with neighboring cells, etc. Their effect on TBI should be considered in terms of time and context. In the same damaged brain tissue, microglia may be in different functional states, which are determined by the expression of molecules such as adhesion, maturation, effector, and among others. The conventional wisdom is that microglia, stimulated by injury, can be activated and polarized into two major phenotypes: M1-like (classical activation) and M2-like (alternative activation).

M1-like activated microglia can express IL-1, HMGB1, TNF-α and other cytokines that promote inflammatory reaction, which is involved in PTE occurrence. In contrast, inflammation mediators secreted by M2-like microglia can downregulate the responses of M1-like microglia, thus potentially inhibiting epileptogenesis. Microglial polarization may be a critical contributor that is directly associated with the pathological prognosis of epileptogenesis. Promoting and maintaining M2-like microglial phenotype after brain injury could be a potential preventive strategy for PTE. However, the current research on M1 and M2-like microglial polarization after TBI is still in the preliminary stage, and the correlation of the findings with epilepsy is even less known. Benson et al. (22) compared microglial polarization in two different acquired temporal lobe SE models, the results showed that pilocarpine-induced SE expressed both M1 and M2 markers, but only M1 markers were upregulated in kainate-induced SE model, which may explain why kainate-induced seizures attack more frequently. Therajaran et al. (23) summarized the possible epileptogenic mechanisms induced by microglial cell polarization, which mainly included altering excitation/inhibition balance, extracellular matrix, oxidative stress regulation, synaptic remodeling and neurodegeneration. In recent years, Hickman et al. (24) have used single-cell sequencing to analyze the gene expression of microglia in aging, and found that microglial polarization representing a mixed and complex state even in the normal physiological aging process. Therefore, oversimplifying microglia polarization into the M1/M2 phenotype does not reflect the microglia/macrophage functional polymorphism in the complex diseases. The occurrence of PTE is result from a combination of multiple factors, and the underlying mechanism remains to be further explored.

### Reactive Astrocytes

Astrocytes are the most widely distributed type of cells in the mammalian brain, and they are not only playing a supporting and isolating role in the CNS, but also participate in pathophysiological processes such as synaptic transmission, neuroimmunity, maintenance of internal environment, and promotion of nerve tissue repair and regeneration (25). Astrocytes play an important role in controlling cerebral blood flow and prevent harmful substances from entering the brain by participating BBB formation with capillary endothelial cells and basal membrane through their terminal foot process. In TBI early stage, astrocytes exert neuroprotective effects by ingesting glutamate(Glu) through glutamate transporters (GLTs), inactivating oxygen free radicals, regulating Na+/K+ balance, and secreting neurotransmitters (26). Within a period after TBI, astrocytes can be activated as reactive astrocytes, which are characterized by high expression of glial fibrillary acidic protein (GFAP), hypertrophy of cell bodies, and extension of primary processes, etc. Astrocytes participate in inflammation through the HMGB1-receptor for advanced glycation end products (RAGE) signaling pathway, thereby activating nuclear factor kappa-B (NF-κB) signaling transduction (27). Reactive astrocytes can also secrete matrix metalloproteinase 9 (MMP9), affecting the integrity of BBB after TBI (28). The glial scar formed by the reactive hyperplasia of glial cells after brain injury, restricts axonal regeneration and functional connections, thereby impeding nerve recovery. Studies have found that astrocytes interact with microglia and other immune cells to produce cytokines, such as insulin-like growth factor 1 (IGF1) and nerve growth factor (NGF), which promote the healing following TBI (29).

Epilepsy has long been thought to be a specific neuronal disease caused by changes within neurons, but novel evidence challenges us to consider that astrocytes also play a non-negligible role in acquired epilepsy. However, the relationship between reactive astrocytes and PTE is unclear. High extracellular potassium and Glu levels after TBI may be the main reasons for seizures induced by altered homeostasis of astrocytes. The homeostasis of extracellular K+ is crucial for regulating neuronal excitability. Kir4.1 is an inward rectifying potassium channel highly expressed in astrocytes of the CNS, buffering excessive spatial potassium load and maintaining the dynamic balance of K+ in the neuronal environment (30). Insufficient K+ buffering and severe seizures were observed in mice with conditional Kir4.1 knockout (31, 32). Similarly, it has been found that TBI injury can lead to loss or down-regulation of Kir4.1 channel of astrocytes (33) and induce PTE (34). A series of studies showed aquaporin-4 (AQP4) and Kir4.1 are co-expressed in astroglial end feet, where Kir4.1 regulates the buffering on extracellular K+ and AQP4 mediates water homeostasis. AQP4 and Kir4.1 co-regulate extracellular interstitial water and electrolyte balance and play a pivotal role in neuronal excitability. Studies have shown that if the number of AQP4 channels is reduced, the ability of Kir4.1 to clear extracellular K+ will be weakened, and the excitability of neurons will be increased, thus triggering seizures (35). The AQP4 and Kir4.1 channels of astrocytes may be new potential targets for the treatment of epilepsy.

Altered neurotransmitter metabolism in astrocytes may also contribute to epileptogenesis. Under normal conditions, the GLTs (mainly GLT1) on astroglial membrane can quickly remove the excess Glu in extracellular space and reduce the excitatory toxicity. Excitotoxic Glu can be converted into non-toxic glutamine (Gn) under the action of glutamine synthetase (GS) after Glu uptake into glial cells. Gn is a substrate for neuronal synthesis of Glu and GABA in neurons. Blocking GS can lead to GABA inhibitory postsynaptic potential. Brain injury results in increased concentrations of extracellular Glu, which can not only overstimulate glutamate receptors such as α-Amino-3-hydroxy-5-methyl-4-isoxazoleproprionic Acid (AMPA) and *N*-methyl-D-aspartate (NMDA), but also affect the function of GLT-1 in astrocytes. Osteen et al. (36) demonstrated that the increased excitability of neurons that survived in injury was related to the long-term activation of NMDA subunit receptors, especially NR2B. Samuelsson et al. (37) discovered that the level of GLT-1 was temporarily decreased in the epilepsy model induced by injecting ferrous chloride, which restricted the uptake of Glu and led to seizures. The results indicated that astrocytic Glu transporter may be one of epileptogenesis mechanism after trauma.

New research point to a direct effect of reactive astrocytes in regulating neuronal function by weakening the inhibition of GABA receptors (38). Two antagonistically acting neuronal chloride (Cl^−^) transporters NKCC1 and KCC2 establish the transmembrane gradient for Cl^−^, which serves as the premise for inhibitory effect of GABA receptors. NKCC1 transports Cl^−^ into the neuron cell body across the membrane to maintain high intracellular Cl^−^, while KCC2 shunts Cl^−^ out of the cell to lower intracellular Cl^−^ concentration. Wang et al. (39) pointed out that NKCC1 expression was up-regulated after TBI, which is responsible for the intracellular Cl^−^ concentration, and gene knockout of NKCC1 or NKCC1 inhibitor bumethanide could reduce seizure frequency. However, a specific KCC2 agonist is not yet available for clinical practice. Besides, long-term chronic epileptic seizures cause astrocytic hyperplasia to participate in hippocampal sclerosis, which will affect the normal physiological regulatory function of brain and play a role promoting epileptogenesis.

### Peripheral Immune Cells

sThe inflammatory response after TBI is not restricted to the CNS. Peripheral cells, such as neutrophils and T lymphocytes, monocytes and macrophages, can infiltrate into the brain through the broken BBB, further complicating the primary injury and local inflammatory response. After activation of CNS resident immune cells, neutrophils are among the first peripheral cellular responders to arrive in the injured brain with just a few hours (40). Neutrophils can permeate through BBB under the induction of cytokines (e.g., TNF-α, IL-1β), chemokines (e.g., CXCL1, 2, 3) and purines, releasing a series of proteases that destroy microvessels and subsequent aggravate BBB destruction. Activated neutrophils are also ROS producer, facilitating oxidative stress and thereby neurodegeneration secondary to TBI (41, 42). While the above data suggest that neutrophils are predominantly detrimental, it is worth noting that neutrophils can also play a beneficial role in promoting neurological recovery after injury. Future research is needed to determine how neutrophils influence wound-healing. Neutrophils have been shown to affect T-cells, including regulatory T cells, CD8+ T cells, and CD4+ T cells, contributing to adaptive immunity (43). In addition, recruitment of neutrophils after TBI is usually accompanied by the arrival of monocytes that turn into macrophages. Macrophages derived from monocytes often participate in the injury response together with yolk-sac derived resident myeloid cells such as microglia, contributing to tissue repairment and even regeneration. However, sustained activation of proinflammatory macrophages is considered to be deleterious, and may lead to progressive neurodegeneration and dysfunction. The mechanism of monocytes recruitment after TBI relies on the production of local chemokine CCL2. Targeting CCL2/CCR2 chemokine signaling pathway can decrease the number of monocytes, which can reduce lesion size and promote neurological recovery (44, 45). CCR2+ mononuclear macrophages infiltration has also been observed in the epileptic tissues (46). Pharmacological inhibition of CCL2 or CCR2 can suppress lipopolysaccharide-induced seizures (47), suggesting an association between monocyte accumulation and seizure susceptibility. As mentioned above, these peripheral immune responses are involved in neuroinflammation. They may promote epileptogenesis following TBI, but how they modulate vulnerability to seizures has not yet been explored.

### Inflammatory Cytokines

**Figure 2:**
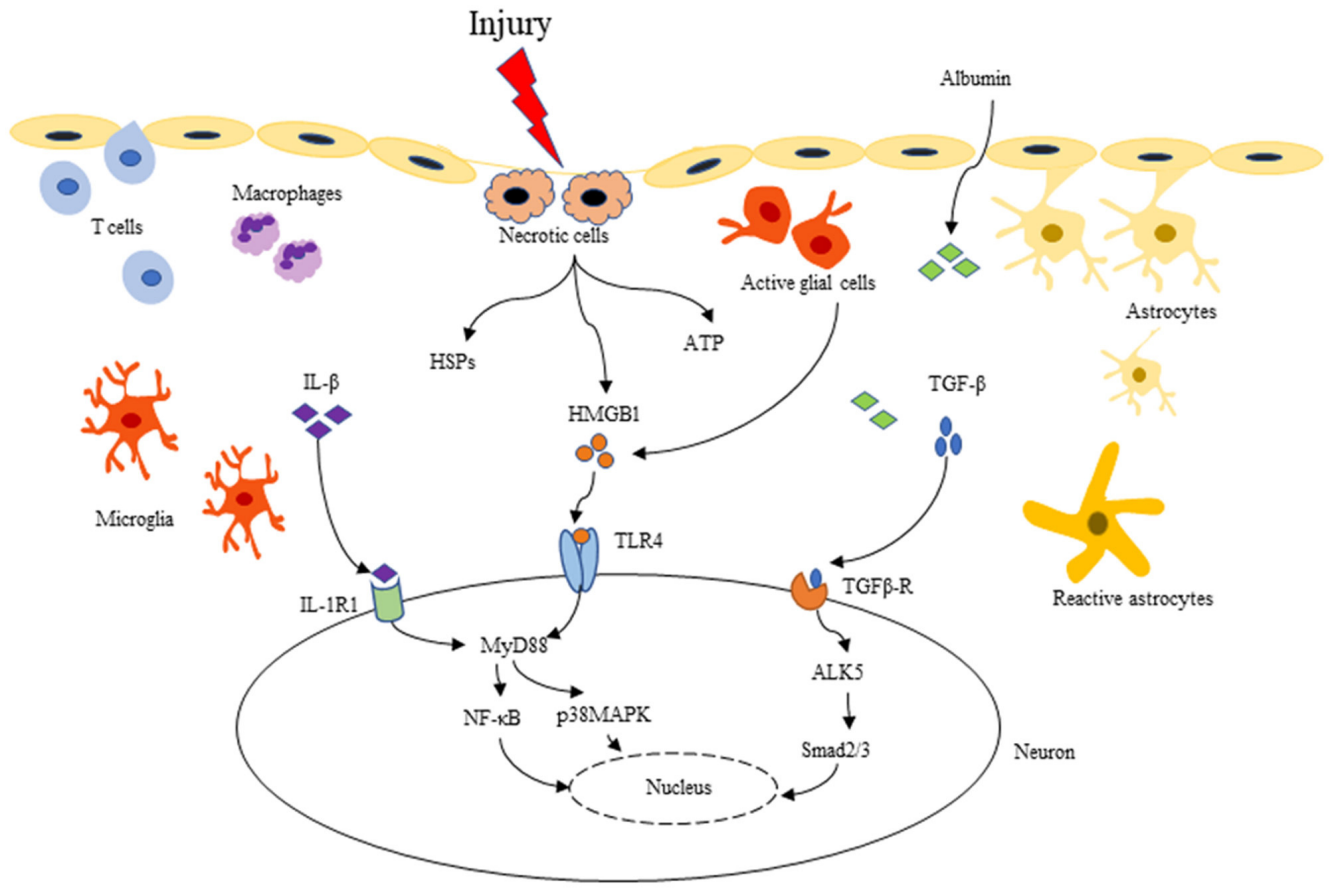
Three key inflammatory signaling pathways related to PTE: IL-1β/IL-1R, HMGB1/TLR4, and TGF-β/albumin pathway. After TBI, BBB is destroyed, as well as microglia and astrocytes are activated. Pro-inflammatory cytokines such as IL-1β, HMGB1 and TGF-β are released into the extracellular matrix. IL-1β binding to IL-1R can activate the downstream NF-κB, p38 MAPK, Src, etc. and initiate intracellular signal transduction through MyD88-dependent or independent signaling pathways. HMGB1 can be passively released by necrotic cells, or be actively secreted to the extracellular from activated microglia and astrocytes, binding to many different types of cell receptors (TLR2/4, RAGE), and activating downstream signaling molecules like the IL-1β/IL-1R signaling pathway. In addition, TBI causes BBB destruction, serum albumin extravasates into the extracellular matrix, activating the TGF-β/ALK5 pathway. These series of inflammatory cascades can lead to increased excitability and synaptic reconstruction, which in turn promotes the development of PTE.

More recently, increasing evidence has supported that neuroinflammation plays a causal role in seizure induction and propagation. TBI gives rise to inflammatory cytokines, mainly including IL-1β, TGF-β, HMGB1, TNF-α, IL-6, and IL-10, which may be the critical inflammatory mediators involved in PTS/PTE (Table 1). These cytokines can recruit neutrophils and monocytes to infiltrate into damaged tissue, expanding the inflammatory cascade reaction (48, 49). Three key signaling pathways that may mediate the relation between neuroinflammation and epileptogenesis: IL-1β/IL-1R signaling pathway, HMGB1/TLR4 signaling pathway, and TGF-β/albumin signaling pathway (Figure 2) (7, 50). These signaling pathways are expected to be more important targets to modulate post-traumatic epileptogenesis.

**Table 1:** Key inflammatory cytokines involved in post-traumatic seizure/epilepsy.

**Factors**	**Fluid/Tissue**	**Time course**	**Role in neuroinflammation**	**Signaling pathways**	**Role in epileptogenesis**
IL-1β	CSF/ ECF Tissue	Peak on day 1–2, decrease on day 2–4. Increased above control 6–122 h after injury.	Pro-Inflammatory: Mediates leukocytes recruitment, other inflammatory factors and chemokines release, glial cells activation, and BBB disruption.	IL-1β/IL-1R Downstream: NF-κB, p38 MAPK, Src, etc.	Pro-epileptogenesis: Increases intracellular calcium [Ca2+]i; Down-regulates GABA (A) receptor function; Inhibits the uptake of Glu through AMPA and NMDA receptors; IL-1R antagonist reduces seizure susceptibility.
HMGB1	CSF	Peak on day 1–3, Decrease on day 4–7.	Pro-Inflammatory: As a typical DAMPs, HMGB1 released passively or actively to cytoplasm or extracellular space; Activates the innate immune system and initiates the inflammatory cascade.	HMGB1/TLR4 Downstream: NF-κB, p38 MAPK, etc.	Pro-epileptogenesis: Regulates long-term enhancement and long-term inhibition; Intracerebral injection of HMGB1 accelerates epileptic activity; Phosphorylates the NR2B subunit of NMDA receptor that promotes calcium influx; Blocking HMGB1/TLR4 decreases both seizure duration and frequency.
TGF-β	CSF	Peak on day 1, gradually decrease over 21 days.	Pro-Inflammatory: Mediates BBB disruption.	TGF-β/albumin	Pro-epileptogenesis: TGF-β can be upregulated in amygdale-kindled or SE models; Down-regulates astrocytes Kir4.1 function; Antagonists of TGF-β receptors can reduce and even inhibit such epileptic activity.
TNF-α	CSF/ ECF Tissue	Peaks early on day 1. Increased above control within 17 min of injury.	Dual role: Activates polymorphonuclear leukocytes; releases ROS and various inflammatory mediators; Damages vascular endothelial cells, and aggravates cerebral edema; Inhibits NMDA-mediated calcium influx; Promotes neurotrophin production.	The TNF-α signaling pathway is mediated by two membrane receptors TNFR1(p55) and TNFR2(p75)	Dual role: The p75 pathway is involved in the anti-seizure activity of TNF-α, whereas the pro-seizure effect is mediated by the p55 pathway; The role of TNF-α signaling pathway in epileptogenesis after TBI remains unclear.
IL-6	CSF/ ECF Tissue	Peak on day 1, decline on day 2–3. Increased above control within 17 min of injury.	Dual role: Increases adhesion molecules and chemokines secretion and enhances leukocyte recruitment; Inhibits the production of TNF-α and reduces NMDA-mediated calcium influx.	-	Dual role: IL-6 can be upregulated after limbic status epilepticus; Over-expression of IL-6 results in seizure threshold reduction; Promotes hippocampal GABAergic neurons loss, leading to an increased propensity for seizures.
IL-10	CSF	Peak on day 1, decline on day 2–3. May have second or third peak of lower magnitude.	Anti-Inflammatory: Inhibits proinflammatory cytokine expression; Reduces leukocyte recruitment and accumulation.	-	Anti-epileptogenesis: Eliminates the hypoxia-evoked epileptiform activity; Renders animals more resistant to FS.

#### IL-1β

Interleukin-1(IL-1) is one of the key mediators involved in both focal and diffuse TBI inflammatory response. The proinflammatory factor IL-1β is the most characteristic member of the IL-1 family and is elevated quickly in damaged brain tissue. IL-1β binding to IL-1R can activate the downstream NF-κB, p38 mitogen-activated protein kinase (MAPK), Src family kinases, etc. Through MyD88-dependent or non-dependent signaling pathways, IL-1β/IL-1R initiates intracellular signal transduction in hippocampal neurons (51). Under physiological conditions, IL-1β is undetectable, which can be upregulated within minutes to hours post-TBI and this high level may last for several months (52). IL-1β is a crucial initiator of the immune inflammatory response, and can involve in leukocytes recruitment, other inflammatory factors and chemokines release (53, 54), glial cells activation, and BBB disruption (55). Frugier et al. (52) found that IL-1β mRNA and protein levels significantly increased in patients who died post-injury, IL-1β neutralizing antibody (56, 57), IL-1R1 antagonist (58) or IL-1R1 gene defect (59) could alleviate TBI-induced glial activation, neutrophil infiltration, brain edema and cognitive dysfunction in animal models of TBI.

Elevated levels of IL-1β in CSF/serum/brain tissue are associated with the development of epilepsy (60). In a previous study, Diamond and his colleagues demonstrated that one of IL-1β functional single nucleotide polymorphisms (SNPs), “rs1143634,” significantly raised PTE risks. In addition, elevated CSF/serum IL-1β ratio was also associated with increased PTE risks (50). In *in vivo* and *in vitro* experiments, IL-1β have been found to enhance the permeability and current strength of Ca^2+^, increase intracellular calcium [Ca^2+^]_I_ (61), down-regulate of GABA (A) receptor function in hippocampal neurons (62) and inhibit the uptake of Glu by astrocytes (63) through AMPA and NMDA receptors. Consistent with these findings, Semple et al. (64) discovered that IL-1R antagonist could reduce seizure susceptibility 2 weeks after the mice injury, accompanied by reduced hyperplasia of hippocampal astrocytes, and spatial memory was improved 4 months later. Other members of the IL-1 family such as IL-1α have also been reported to up-regulate in brain tissue after TBI (65), but are not related to the prognosis of TBI or epilepsy. Taken together, these data support a key role for IL-1β in epileptogenesis, and implicate IL-1β/IL-1R signaling pathway as a potential target to prevent PTE.

#### HMGB1

TBI causes the release of DAMPs, such as HMGB1, ATP, heat shock proteins (HSPs), and S100. By interacting with PRRs, or RAGE, DAMPs act to activate intracellular signal transduction and initiate an inflammatory cascade. HMGB1 is a multifunctional protein whose function depends on its subcellular localization. In the nucleus, it plays the role of stabilizing nucleosomes, participating in gene transcription, and regulating DNA replication and repair. As a typical DAMPs, HMGB1 can be passively released by necrotic cells, or be actively secreted to the extracellular after tissue injury, activating the innate immune system. TLR4 is the hypothesized receptor of HMGB1 and mainly distributed in neurons and glial cells. Here, we particularly focus on HMGB1/TLR4 axis which recently are known to be implicated in TBI-induced immune inflammatory response and epileptogenesis.

Recent studies have indicated that HMGB1 can regulate long-term enhancement and long-term inhibition of the hippocampus after activation of TLR and RAGE (66) and play a role in synaptic transmission and plasticity of neurons, which may be relevant to epileptogenesis and cognitive dysfunction. Kainate and bicuculline-induced acute and chronic epileptic model showed that HMGB1 was highly expressed in neurons and glial cells at the injured sites of mice, and could be transferred from the nucleus to the cytoplasm and then secreted to the extracellular space, suggesting that it might be involved in the initiation of epilepsy (67). New studies have noted that the ATP-gated ionophilic P2X7 receptor promotes the release of IL-1β and HMGB1 from glial cells by mediating the activation of NALP3 inflammasome, thereby facilitating seizures (68). The direct evidence of HMGB1 involved in PTE was that intracerebral injection of HMGB1 in mice accelerated the acute epileptic activity induced by convulsive drugs and increased the frequency and severity of seizures. HMGB1/TLR4 antagonist or anti-HMGB1 monoclonal antibody significantly increased the epileptogenic threshold, decreased both seizure duration and frequency (67, 69). A study using primary cultured hippocampal neurons found that TLR4 mediated HMGB1 signaling phosphorylates NR2B subunit of NMDA receptor and promotes calcium influx, which is pivotal for inducing excitotoxicity and accelerating epileptogenesis (70). The HMGB1/TLR4 signaling pathway is similar to that of IL-1β/IL-1R, which activates the downstream NF-κB and MAPK through the MyD88-dependent or independent pathways, and promotes the neurovascular dysfunction. Inhibiting TLR-4 can reduce brain edema and IL-6 production post-TBI (71). Overall, there is much accumulating evidence implicating HMGB1/TLR4 signaling pathway in epileptogenesis, however further studies are needed to prove their precise mechanism in PTE.

#### TGF-β

TGF-β is a multifunctional cytokine involved in many different cellular processes such as cell proliferation, differentiation, adhesion, migration and apoptosis (72, 73). TGF-β can be up-regulated in various CNS diseases, including multiple sclerosis, Alzheimer's disease (AD), stroke, and TBI. Clinical studies found that TGF-β in CSF was significantly elevated 24 h after TBI, and gradually recovered after 21 days, and the level of TGF-β was related to the function of BBB (74). TGF-β is mediated by two TGF-β receptors (TGFβR I and TGFβR II), which phosphorylates downstream Smads protein (Smad 1/5 or Smad 2/3) through ALK1 and ALK5 receptors, activates NF-κB or MAPK, and regulates target gene transcription.

Is TGF-β associated with epileptogenesis? Animal experiments have shown that TGF-β upregulation in neurons of amygdale-kindled rats (75) and in hippocampal astrocytes of SE models (76), supporting the potential role of TGF-β in epileptogenesis. BBB dysfunction is a hallmark of brain injury. TGF-β has also been demonstrated to be involved in microglial activation (77) and pericyte-induced BBB function (78). Increased BBB permeability was found in MRI of PTE patients, which was consistent with the site of epileptogenic foci (79). Studies on the ultrastructure of surgically resected epileptic tissues have confirmed significant anatomical abnormalities in BBB components, including endothelial cells, basement membranes, and tight junctions (80). van Vliet et al. (81) found a positive correlation between BBB permeability and seizure frequency in the chronic epileptic model, suggesting that BBB dysfunction is conducive to the development of temporal lobe epilepsy (TLE). *In vivo* and *in vitro* experiments (82) have confirmed that exosmic serum albumin post-injury could activate TGF-β/ALK5 pathway of astrocytes, and TGF-β inhibition, SJN2511, could effectively reduce and prevent synaptic remodeling and seizures. Consistent with this finding, Iven et al. (83) also found that TGF-β1 was directly exposed to albumin after BBB destruction, which could cause local cortical dysfunction and induce epileptic discharge. One possible mechanism is local BBB damage leading to serum albumin seep into the cerebral cortex microenvironment. Albumin is uptake by astrocytes through its TGFβR II, and then down-regulate membrane Kir4.1, resulting in an increase in extracellular potassium, which leads to excessive activation of NMDA receptors and causes neuronal hyperexcitability and epileptiform discharge. Antagonists, blocking albumin binding to TGF-β receptors, have been reported to reduce or inhibit such epileptic activity (83). Transcriptome analysis revealed that TGF-β1 induced a similar transcriptional regulation patterns when exposed to serum albumin; Blocking the TGF-β signaling pathway not only reversed the transcriptional response after albumin exposure, but also prevented epileptic activity (84). Therefore, damage to microvessels during TBI may lead to serum albumin extravasation and inflammatory response, which are key steps in PTE development.

#### TNF-α

TNF-α, the primary subtype of TNF, is one of the critical mediators involved in immune response and neuroinflammation in the CNS, and is known to be released acutely after tissue injury in an active fashion by reactive glial cells, neurons and vascular endothelial cells. TNF-α can activate polymorphonuclear leukocytes, release ROS and various inflammatory mediators, play an important role in secondary brain injury. In addition, TNF-α can directly damage vascular endothelial cells, cause microvascular spasm, increase capillary permeability, and aggravate cerebral edema (85). The TNF-α signaling pathway is mediated by two membrane receptors TNFR1 (also called p55) and TNFR2 (also called p75). TNFR1, which is widely expressed, can be activated by binding to soluble TNF (solTNF) or transmembrane TNF (tmTNF) mediating downstream signaling pathways to initiate apoptosis (86). Compared with TNFR1, TNFR2 expression is limited and mainly released by microglia and endothelial cells known to regulate cell proliferation (87). Current studies have suggested that TNF-α may play a dual role as a pro-inflammatory and anti-inflammatory cytokine, depending on the timing and signaling cascade involved. Scherbel et al. (88) found in TNF-α knockout mice that TNF-α produced in the early stage of TBI may be deleterious, but the lack of TNF may increase neuronal loss and recovery time in the chronic period. TNF-α and IL-1 stimulate astrocytes to produce NGF and IL-10, which may be the basis of the neuroprotective and anti-inflammatory effects of TNF-α (89, 90).

As for the demonstration of a dual role of TNF-α in neuroinflammation, studies have shown that TNF-α has both epileptogenic and antiepileptic effects. In one study, transgenic mice with neuronal overexpression of TNF-α developed seizures and early death (91). Another study demonstrated that mice lacking TNFα receptors were observed to have prolonged seizures (92). The dichotomous role of TNF-α in seizures is thought to be mediated by the different receptors, p55 and p75. Balosso et al. suggested that the p75 pathway is involved in the anti-seizure activity of TNF-α, whereas p55 pathway mediates the pro-seizure effect. However, the mechanism that determines the predominance of these two pathways has not yet been explored, the role of TNF-α signaling pathway in epileptogenesis after TBI remains unclear.

#### IL-6

IL-6 is a multifunctional factor that can be secreted by several cells in the CNS, including microglia, astrocytes and neurons, and may also play a dual role in neuroinflammation following TBI. IL-6 has been reported to increase adhesion molecules and chemokines secretion, enhancing leukocyte recruitment and acting as a pro-inflammatory cytokine. In contrast, IL-6 can inhibit the production of TNF-α, and reduce NMDA-mediated calcium influx. Swartz et al. (93) showed that IL-6-deficient mice were found to have a slowed healing process following TBI, whereas overexpression of IL-6 resulted in a more rapid recovery by improving re-vascularization of the injury site.

Similarly, there is some evidence also implicated a role for IL-6 in seizure pathologies.

IL-6, IL-1β, and TNF-α were rapidly upregulated 2 h after limbic SE induced by electrical stimulation, peaking at 6 h, which may cause hyperexcitability in epileptic tissue (94). IL-6 was also upregulated in the CSF of patients with newly diagnosed tonic-clonic seizures (95). Elevated IL-6 in the CSF or plasma has been reported to be associated with the severity of epileptic seizures. Transgenic mice over-expression of IL-6 resulted in seizure threshold reduction and hippocampal excitation augment. In fact, IL-6 et al. inflammatory cytokines may promote hippocampal GABAergic neuron loss, leading to an increased propensity for seizures owing to reduced inhibitory interneurons (96).

#### IL-10

The cytokine IL-10 is a potent anti-inflammatory cytokine, which is found in the CSF of patients with TBI. Intravenous injection of IL-10 significantly reduced proinflammatory cytokine expression (particularly TNF-α and IL-1) and improved neurological outcome in lateral FPI model of rats (89). It has been indicated that IL-10 plays a neuroprotective role by acting on the peripheral immune system and is associated with circulating monocytes which can inhibit leukocyte recruitment and accumulation.

Although few studies have shown that IL-10 regulates susceptibility to seizures after TBI, several animal studies have indicated its anti-seizure effects. For example, IL-10 application was shown to eliminate the hypoxia-evoked epileptiform activity in rat hippocampal slices (97). Another study suggested that IL-10 was genetically related to febrile seizures (FS) in rats, the seizure threshold temperature in IL-10 treated rats was higher than that in control groups, indicating that IL-10 made animals more resistant to FS (98). The antiepileptic functions of IL-10 are thought to be due to the anti-inflammatory effects of cytokines.

### Chronic Neuroinflammation

About a quarter of TBI patients develop progressive neurodegenerative syndromes such as AD, chronic traumatic encephalopathy (CTE), and PTE. The underlying pathogenesis remains unclear, but inflammation has received increased attention from researchers in recent years concerning the pathophysiologic mechanism of various neurodegenerative conditions. As we all know, the pathogenesis of AD is still controversial. There are several hypothetical mechanisms, such as Aβ cascade reaction, Tau hyperphosphorylation, cholinergic hypothesis, etc. However, several lines of evidence suggest that chronic neuroinflammation caused by brain trauma may be a potential factor for AD. Studies in AD models have suggested that neuroinflammatory cytokines and reactive microglia can promote the accumulation and deposition of pathological tau, which may explain the relationship between TBI-induced inflammation and the predisposition to AD (99, 100). But how Aβ induces neuronal hyperexcitability is still unknown (101, 102). Ren et al. (103) explained the possible mechanism through whole-cell recordings of mouse brain slices. They found that Aβ promotes dopamine release in the anterior cingulate cortex, overactivating D1 receptors on interneurons which inhibits GABA release, and then leading to excitatory/inhibitory imbalance. In addition, the accumulation of Aβ has been shown to induce microglial activation and pro-inflammatory mediators release (104). The production of Aβ toxicity after TBI and the disruption of neurotransmitters such as dopamine may have an impact in the development of PTE through inflammatory mechanisms, but this question remains to be further explored. CTE is a progressive neurodegenerative disease associated with repeated head injury, and is most common in athletes and soldiers (105). Studies have shown that activated microglia can last for several years after brain injury, suggesting that a role for a persistent TBI-induced neuroinflammation in CTE development (106). Aungst et al. (107) found that chronic inflammation caused by repeated mild traumatic brain injury (mTBI) can change hippocampal synaptic plasticity, leading to sustained cognitive and neuropsychiatric changes.

In the Kainic acid (KA)-induced acute epilepsy model, the hyperphosphorylated Tau was significantly increased, and the time and location of Tau were consistent with that of mossy fiber sprouting (108). Recent studies have demonstrated that late-onset seizures after TBI are also accompanied by a certain degree of neuronal degeneration and hyperphosphorylated tau (109). Neurofibrillary tangles (NFTs) consisted of Tau were also found in surgical specimens of patients with refractory epilepsy and focal cortical dysplasia, and these Tau tangles are specifically located in the dysplastic area (110, 111). Protein phosphatase 2A (PP2A) appears to be a major serine/threonine protein phosphatase that plays a negative regulatory role in signal transduction, and its increased activity can promote the dephosphorylation of hyperphosphorylated tau (112).

Studies (113) have reported that selenate specifically targets hyperphosphorylated tau, enhances PP2A activity and inhibits seizures in multiple epileptic animal models, suggesting that this may be a new approach to the treatment of PTE. Hippocampal sclerosis is associated with tau protein degeneration in patients with PTE (109). However, there is still no definitive proof showing the hyperphosphorylated tau-based mechanisms in PTE. A comprehensive understanding of the relationship between chronic neuroinflammation and PTE will require more research or more advanced neuroimaging techniques (such as PET imaging) that enable us to study the potential mechanisms of Aβ deposition, tau phosphorylation and microglia/astrocytes activation in neurodegenerative diseases post-TBI.

## Therapeutic Targets

Although neuroinflammation is increasingly recognized as a critical mechanism in the development of epilepsy, few studies have been conducted on immune-targeted pharmaceuticals of PTE to date. A phase IIA clinical trial showed that the selective IL-converting enzyme (ICE)/Caspase 1 inhibitor VX-765 could effectively alleviate seizures in some patients and continue for a period of time after drug discontinuation (114). Anakinra, an IL-1R1 antagonist, has also been demonstrated to reduce refractory epilepsy (115). The broad-spectrum antibiotic minocycline was reported to inhibit the microglial activation and proinflammatory factors release, reducing the frequency of seizures in patients with drug-resistant epilepsy (116). Given the complex, and variable inflammatory pathways associated with, combinations of anti-inflammatory drugs may be more effective than a single medication. VX-765 and TLR4 antagonist therapy on the IL-1R1/TLR-4 signaling pathway effectively prevented the epilepsy progression and significantly reduced the chronic seizures (117). Similarly, Kwon et al. (118) found in the pilocarpine-induced SE model that a combination of COX2 inhibitor CAY10404 and minocycline was more effective than single drug in reducing neuron damage of the hippocampus CA1 region and spontaneous seizures. As mentioned above, the TGF-β/albumin signaling pathway has also generated interest as an immune-therapeutic target for PTE. Studies have reported that angiotensin II type 1 receptor antagonist losartan can effectively block the TGF-β activation induced by albumin, delay the development of acquired epilepsy and reduce the severity of seizures (119). Inducible nitric oxide synthase (iNOS) is a key mediator of immune activation and inflammation, and its inhibitor, 1,400 W, has shown to inhibit epileptogenesis in rodent models of epilepsy (120–122). It is important to note that some of the drug-resistant epilepsy therapies, such as non-steroidal anti-inflammatory drugs, steroids, cannabinoid drugs, ketogenic diet and vagus nerve stimulation, have also been proved to have an anti-inflammatory effect, but there is still a notable lack of conclusive evidence to delineate these relationships (123, 124). Anti-epileptic therapeutics targeting immune inflammation has shown great potential in preventing and treating PTE, which is worthy of further research.

## Conclusions

PTE is a severe complication of TBI, which significantly affects the quality of life of patients. As PTE is drug-resistant in at least one-third of patients, further research is needed to find novel therapeutic strategies for preventing the development of epilepsy after TBI. Clinical and experimental evidence has emphasized brain neuroinflammation as a key factor contributing to epileptogenesis. This review presents our current understanding of the immune inflammatory response to PTE, including microglial activation, reactive astrocytes proliferation, peripheral immune cells infiltration, inflammatory cytokines release, chronic neuroinflammation and potential therapeutic targets. However, the pathogenesis of PTE is very complex and has not yet been fully elucidated. There are still many unknown areas worth exploring further. One of the most essential areas warranting investigation is the possible inflammatory signaling pathways, especially the TGF-β/albumin signaling pathway. Another field of concern is the relationship between neurodegeneration and PTE. Inhibiting tau phosphorylation by sodium selenite may be a new approach to the treatment of delayed seizures. A better understanding of how the inflammatory response promotes epileptogenesis after TBI is the key to immune-targeted therapy.

## Author Contributions

LS conducted literature review and wrote the initial draft of the manuscript. WS, HY, and RL made preliminary revision. JW and QW made critical revision. All authors contributed to manuscript revision and approved the submitted version.

## Conflict of Interest

The authors declare that the research was conducted in the absence of any commercial or financial relationships that could be construed as a potential conflict of interest.

## Abbreviations

AD: Alzheimer's disease
AEDs: Antiepileptic drugs
AMPA: α-Amino-3-hydroxy-5-methyl-4-isoxazoleproprionic Acid
AQP4: Aquaporin-4
BBB: Blood-brain barrier
CNS: Central nervous system
CSF: Cerebrospinal fluid
CTE: Chronic traumatic encephalopathy
DAMPs and PAMPs: Damage- or pathogen-associated molecule patterns
FPI: Fluid-percussion injury
FS: Febrile seizures
GFAP: Glial fibrillary acidic protein
GLT: Glutamate transporter
Glu: Glutamate
Gn: Glutamine
GS: Glutamine synthetase
ICE: IL-converting enzyme
IGF1: Insulin-like growth factor 1
IL-1: Interleukin-1
KA: Kainic acid
MAPK: Mitogen-activated protein kinase
MHC: Major histocompatibility complex
MMP9: Matrix metalloproteinase 9
mTBI: mild traumatic brain injury
NFTs: Neurofibrillary tangles
NF-κB: Nuclear factor kappa-B
NGF: Nerve growth factor
NLRs: Nucleotide-binding oligomerization domain like receptors
NMDA: N-methyl-D-aspartate
NO: Nitric oxide
PP2A: Protein phosphatase 2A
PRRs: Pattern recognition receptors
PTE: Post-traumatic epilepsy
PTS: Post-traumatic seizure
RAGE: Receptor for advanced glycation end products
ROS: Reactive oxygen species
SE: Status epilepticus
SNPs: Single nucleotide polymorphisms
TBI: Traumatic brain injury
TLE: Temporal lobe epilepsy
TLRs: Toll-like receptors.
